# Clinical profile and outcomes of pediatric hypertrophic cardiomyopathy in a South Indian tertiary care cardiac center: a three decade experience

**DOI:** 10.1186/s12887-023-04255-z

**Published:** 2023-09-07

**Authors:** Gousia Mukhtar, Bijulal Sasidharan, Kavassery Mahadevan Krishnamoorthy, Harikrishnan K. N. Kurup, Arun Gopalakrishnan, Deepa SasiKumar, Sankara Sarma P, Ajit Kumar Valaparambil, Sivasankaran Sivasubramonian, Harikrishnan Sivadasanpillai

**Affiliations:** 1 Department of Cardiology, Sree Chitra Tirunal Institute for Medical Sciences and Technology, SCTIMST, Thiruvananthapuram, Kerala 695011 India; 2 Achutha Menon Center for Health Science Studies, Thiruvananthapuram, Kerala 695011 India

**Keywords:** Pediatric, Hypertrophic cardiomyopathy, Outcomes, India

## Abstract

**Introduction:**

Although much research has been done on adult hypertrophic cardiomyopathy, data on pediatric hypertrophic cardiomyopathy is still limited.

**Methods and results:**

The study enrolled all patients with cardiomyopathy who presented to us between 1990 to 2020 and were younger than 18 yrs. During the thirty-year study period, we identified 233 cases of pediatric cardiomyopathy. Sixty-three cases (27%) had hypertrophic cardiomyopathy. Out of the 63 HCM cases, 12% presented in the neonatal period and 37% presented in the first year of life. The median age of presentation was 7 yrs (Range 0.1–18 yrs). Sixteen patients had proven syndromic, metabolic, or genetic disease (25%). LV outflow obstruction was present in 30 patients (47%). Noonan syndrome was present in 9 of the 63 patients (14%). Dyspnea on exertion was the most common mode of presentation. Cardiac MRI was done in 28 patients, out of which 17 had late gadolinium enhancement (LGE). Mid myocardial enhancement was the most common pattern. Four patients had LGE of more than 15%. Over a mean follow-up period of 5.6 years (0.1–30 years), twenty-one were lost to follow-up (33%). Among the patients whose outcome was known, eleven died (26%), and thirty-one (73%) were alive. The 5-year survival rate of HCM patients was 82%, and the 10-year survival rate was 78%. Seven died of sudden cardiac death, three from heart failure, and one from ventricular arrhythmias. Sustained ventricular arrhythmias were seen in three patients and atrial arrhythmias in two. First-degree AV block was seen in 10 patients (15%) and bundle branch blocks (BBB) in five (8%). Eight patients required ICD or transplant (12.7%). Two patients underwent ICD for primary prevention, and one underwent PPI for distal AV conduction disease. Among the various clinical, echocardiographic, and radiological risk factors studied, only consanguinity showed a trend towards higher events of death or ventricular arrhythmias (*P*-value 0.08).

**Conclusion:**

More than one-third of our HCM cohort presented in infancy. LV outflow tract obstruction is common (47%). Mid myocardial enhancement was the most common pattern of late gadolinium enhancement. SCD was the most common cause of death. The outcome in our HCM cohort is good and similar to other population cohorts. Only Consanguinity showed a trend towards higher events of death or ventricular arrhythmias.

## Introduction

Although much research has been done on adult HCM, research on pediatric cardiomyopathy, is surprisingly scarce. Pediatric onset HCM has diverse etiologies with their variable respective outcomes. Predictors of death or adverse outcomes in pediatric-onset HCM remain unresolved, challenging risk stratification. Nugent et al. reported on 80 children with hypertrophic cardiomyopathy from the National Australian Childhood Cardiomyopathy Study [1]. Yetman et al. published a study on long-term outcome and prognostic determinants in 99 children With hypertrophic Cardiomyopathy in 1998 [2]. Maruizi et al. assessed 100 patients with pediatric-onset hypertrophic cardiomyopathy diagnosed in national referral centers for cardiomyopathies in Italy [3]. Since data on pediatric cardiomyopathy from our part of the world is still limited, this study aimed at describing the clinical profile and outcomes of pediatric hypertrophic cardiomyopathy in our population as well as to analyse the predictors of outcome.

## Materials and methods

The study enrolled all patients with cardiomyopathy who presented to us between 1990 to 2020 and were younger than 18 yrs. After taking informed consent, we reviewed the available medical records of each enrolled patient. This retrospective cohort of children was prospectively followed up to study their outcome and the possible predictors of outcome. Exclusion criteria were congenital heart defects not associated with malformation syndromes, abnormal ventricular size or function ascribed to intense physical training, chronic hypertension and maternal gestational diabetes mellitus. Routine genotyping for sarcomeric protein mutations and Noonan syndrome was unavailable during most of the study period. After directly reviewing all available cardiac information, we assigned each patient to a diagnostic category according to the phenotypic characteristics following the European Society of Cardiology Classification [4]. Cardiomyopathies were defined as abnormalities of the ventricular myocardium unexplained by abnormal loading conditions or congenital heart disease [5]. Hypertrophic cardiomyopathy was characterized by otherwise unexplained septal hypertrophy, left ventricular free-wall hypertrophy, or both (wall thickness more than 2 SD above the normal of mean for BSA) [6]. The need for ICD was based on one or more of these major risk factors: Family history of HCM and/or SCD, NSVT on an ambulatory monitor, massive LVH, or unexplained syncope [7]. Right ventricular involvement was defined by a right ventricular free wall thickness > 4 mm in the absence of pulmonary valve stenosis [8]. The morphology of left ventricular involvement was classified as either asymmetric septal hypertrophy if the interventricular septum was predominantly involved or concentric left ventricular hypertrophy if both the left ventricular free wall and interventricular septum were affected to a similar degree [1]. Left ventricular outflow tract obstruction was defined as measured resting or provoked systolic peak instantaneous gradient of > 30 mm Hg on echocardiography [7]. Noonan syndrome was diagnosed if characteristic phenotypic features were identified on clinical examination. Familial hypertrophic cardiomyopathy was defined as an affected first- or second-degree relative in the absence of Noonan syndrome, a mitochondrial disorder, or metabolic condition [1]. Massive left ventricular hypertrophy (LVH) was defined for patients younger than 12 years as the left ventricular maximal wall thickness of more than 20 SD; the adult cutoff value of 30 mm was used for all participants older than 12 years [7]. Massive LVH have been variously defined in pediatric HCM literature with some studies considering z score of more than 6 as massive LVH and others defining it as that more than 20. The end-stage phase of HCM was defined as an LV ejection fraction less than 50% [9].

Collected data include demographic descriptors, relevant history, examination findings, and laboratory data. Patients were considered on follow-up if they were seen in the out-patient department in the previous six months. If not seen within the last six months, patients or their guardians were reached out by telephonic interview. A single observer read the earliest available ECG and converted the measurements to age-appropriate Z scores [10]. QT interval (QTc) dispersion was calculated manually from a 12-lead ECG as the largest difference in QTc intervals between leads. Echocardiographic measurements of left ventricular dimensions, diastolic free wall, and septal thickness were expressed as Z scores based on body surface area [11]. The outcome parameters to be studied were Age at Death, Circumstances of death (SCD, Death from Ventricular Arrhythmia, or Death from Heart failure), AV block, BBB, and Requirement of ICD/transplant.

### Statistical analysis

The data analysis was performed using the SPSS Statistics software for Windows Version 21. Continuous variables were expressed as either mean, standard deviation, or median, depending on the overall variable distribution. Descriptive summaries were presented as frequencies and percentages for categorical data. Continuous variables were compared using Student's t-test or Mann–Whitney U test as appropriate. Group comparisons were made using χ2 tests.

## Results

During the thirty-year study period (1990–2020), we identified 233 cases of pediatric cardiomyopathy, out of which sixty-three subjects had hypertrophic cardiomyopathy (27 percent). The demographic characteristics of the patients are described in Table 1. The median age of presentation of HCM patients was 7 yrs (Range 0.1–18 yrs).

**Table 1:** Depicts the clinical profile of 63 HCM patients

Character (*n* = 63)	Frequency (Percentage)
Age	
< 1 month	8 (12)
1 m—12 months	16 (25)
> 1 yr -12 years	25 (39)
> 12 yrs	14 (22)
Males	38 (60)
Mode of presentation	
Dyspnea on exertion	21 (33)
Incidentally	20
Screening	8
Syncope	10 (15)
Angina on exertion	2
Antenatally diagnosed	1
Consanguinity (*n* = 55)	5 (9)
Syncope	18 (29)
Family H/O Cardiomyopathy	18 (29), Not known in 2
Family H/O SCD	14 (22)
Syndromic	15 (24); Noonan Syndrome Most common
NYHA / Ross FC	
I	16 (25%)
II	44 (69%)
III	3 (4.7%)
Cardiac examination	
Cardiac enlargement	8 (12)
LVS3	4 (6)
Loud P2	8 (12)
S4	24 (38)
ESM	47 (74)
Double apical impulse	10 (15)
CXR (*n* = 54)	
CTR (m ± SD)	0.57 ± 0.07 (0.45–0.8)
RAE	15 (27)
LAE	35 (64)
PVH	24 (44)

*SCD* Sudden cardiac death, *RAE* Right atrial enlargement, *LAE* Left atrial enlargement, *PVH* Pulmonary venous hypertension, *NYHA* New York Heart Association, *CTR* Cardiothoracic ratio, *CXR* Chest X-ray, *ESM* Ejection systolic murmur

### Presenting symptoms and history

The most common mode of presentation was dyspnea on exertion (33%). Ten patients presented with syncope (15%). A history of consanguinity was known in 55, out of which five patients (9%) had a history of parental consanguinity. Syncope developed at any time during the course in 18 patients (29%). Family History of HCM was present in 18 (29%), and family history of sudden cardiac death was present in 14 patients (22%).

### Syndromic, metabolic, and genetic disease

Sixteen patients had proven syndromic or metabolic disease (25%). Noonan Syndrome was the most common syndrome found (9 patients). One patient had Friedrichs Ataxia (GAA mutation positive). Pompes disease was suspected in six, out of which three were tested for the mutation, and among them, one tested positive (alpha-glucosidase mutation). This child also had an associated short PR interval and received enzyme replacement. One patient with underlying Childhood-onset myoclonus dystonia tested positive for MYBPC3 mutation. Three had unclassified syndromes.

### Electrocardiography

ECG characteristics are described in Table 2. Ten patients (15%) had first-degree AV block, PR interval > 2.5 z score. The median QT interval in these patients was 453 ms (Range 400 ms-588 ms). ECG was available for reinterpretation in 41 patients. QT dispersion was 52 (Range 20–126). Pre excitation was present in two subjects, one of whom underwent an electrophysiological study which showed mid septal accessory pathway and no inducible VT. He was kept on medical follow-up given intermittent preexcitation. There were no other clinical features that would suggest any syndromal association in both subjects. Genetic studies were not done because of financial constraints.

**Table 2:** ECG features of HCM cohort patients

ECG (*n* = 59)	
PR interval, Median(Range)	140 (80–250)
PR interval z score, Median(Range)	1.0(- 3.2 – 7.27)
PR interval > 2.5 z score, n(%)	10 (15)
QRS d, Median(Range)	100 (80–180)
QRS d zscore, Median(Range)	2.0 ( -1.0 – 8.3)
QRS d > 2.5 z score, n(%)	5 (8)
QT interval, ms Median(Range) (*n* = 41)	453 (400–588)
QT dispersion (*n* = 41)	52 (20–126)
LAD, n(%)	20 (32), 2 had NW axis
RAD, n(%)	2 (3)
Normal axis, n(%)	37 (62)
q waves, n(%)	37 (62)
ST depression (*n* = 41)	16 (39)
LVH, n(%)	53 (89)
Preexcitation	2

*LAD* Left axis deviation, *RAD* Right axis deviation, *NW axis* Northwest axis, *LVH* Left ventricular hypertrophy

### Echocardiography

Echocardiographic characteristics are presented in Table 3. LVOT Obstruction is present in 30 patients (47%), 23 had gradient > 50 mmHg. Five had concentric left ventricular hypertrophy, and the rest had asymmetric septal hypertrophy. The median interventricular septal thickness z score (*n* = 40) is 4.3 (Range 2.33–7.3). Only one of our patients had massive left ventricular hypertrophy on echocardiography. The median LVOT gradient on echocardiography was 88 mmHg (Range 15–169 mmHg. One patient had apical HCM. Right ventricular outflow obstruction in the absence of valvular stenosis was seen in one patient. We assessed diastolic dysfunction in 30 patients, and eight had moderate or severe diastolic dysfunction. Mean Deceleration time (*n* = 8)—172 ms (Range 70 -290), E/A ratio (*n* = 23)—1.35 (0.22–2.4). One child with infantile-onset of obstructive HCM had documented infective endocarditis of the mitral valve.

**Table 3:** Echocardiographic features of HCM patients

ECHO	Median (Range)
IVSs (mm)	16 (7–36)
IVSs z score (*n* = 40)	4.31 (2.33 – 7.3)
PWs (mm)	15 (6–38)
PWs (z score) (*n* = 40)	2 ( -1.49 – 8.06)
LA dimension, mm (mean)	30 ± 12 (12–80)
LA dimension z score (*n* = 40)	2.56 (-1.08—8.9)
LVEF, mean	71 ± 12 (30–87)
< 50, n	4
50–65, n	8
> 65, n	51
LVOT Obstruction, n(%)	30 (47)
LV AO gradient, mmHg Median, (Range)	88 (15–169)
30–50, n	7
50–100, n	13
> 100, n	10
SAM	41 (64)
PAH	5 (7.9)
Mitral regurgitation	
No	33 (52)
Mild	17 (26)
Moderate	11 (17)
Severe	2 (3)
Biventricular	5
Apical	1
Burned out HCM	4

IVS s Interventricular septum thickness in systole, PW s posterior wall thickness in systole, LA left atrial diameter, LVOT left ventricular outflow tract, LV AO Left ventricle to aorta gradient, SAM Systolic anterior motion, PAH Pulmonary artery Hypertension, LVEF Left ventricle ejection fraction

### End-stage HCM

Four children proceeded to burned out phase in this series. The essential characteristics of these children are shown in Table 4. None of them had history of parental consanguinity. Only one had family history of SCD. Three of them had nonobstructive HCM, and only one had severe obstruction with a gradient > 100 mmHg. Two presented with syncope. All subjects with burned-out HCM underwent CMR and had late gadolinium enhancement. Three had mid myocardial enhancement, and one had diffuse LGE. One of them had LGE > 15%. Two patients had first degree AV block, one had RBBB, and one had 2:1 AV block.

**Table 4:** Characteristics of end-stage HCM patients

Patient number	Age at onset, years	Age at ES recognition, years	Syncope	LVOTO	VT	CMRI LGE	Outcome
1	**8**	**17**	**N**	**Y**	**Y**	**Y**	**Lost to follow up**
2	**Not known**	**8**	**Y**	**N**	**Y**	**Y**	**Death due to incessant VT at 13 yrs age**
3	**11**	**17**	**Y**	**N**	**N**	**Y**	**Lost to follow up, Underwent PPI for 2:1 AV block, HV Block on EPS**
4	**1**	**15**	**N**	**N**	**N**	**Y, > 15%**	**SCD within four months of ES recognition**

*VT* Ventricular Tachycardia, *CMRI LGE* Cardiac MRI Late gadolinium enhancement, *SCD* Sudden cardiac death, *ES* End Stage, *EPS* Electrophysiological study, *PPI* Permanent pacemaker Implantation

### Cardiac catheterization

Hemodynamic and other clinical features are described in Table 5. Cardiac catheterization was done in 17 patients. Median LV to Aorta gradient by cath was 100 mmHg (Range 30–140). Coronary artery abnormality was identified in two patients. One had a right coronary artery arising from the left sinus with a slit-like ostium and intramural course of RCA. One had intramyocardial mid and distal left anterior descending coronary artery.

**Table 5:** Hemodynamic features and other clinical characteristics of the HCM cohort

Holter (30)	
Normal	21
NSVT/ VPCs	5
Atrial tachycardia/AFib	2
Conduction abnormalities (CHB / BBB)	2
CMRI (28)	
LGE present	17
Midmyocardial	6
Subendocardial	3
Diffuse	6
LGE Absent	11
Medications	
Beta-blocker	48
Metoprolol	29
Propranolol	12
Bisoprolol	3
Atenolol	4
Diuretics	8
Digoxin	1
ACE Inhibitor	1
Amiodarone	2
Diltiazem	1
NT pro BNP pg/ml, Median(range) (*n* = 12)	2052 (40–9540)
Cardiac cath (*n* = 17)	
PA mean (mmHg)	22 ± 6
LVEDP,mean (mmHg)	15 ± 7
LV AO grad by cath, mmHg (median) (*n* = 9)	100 (30–140)
Brokenbrough Braunwald sign	7 (77)

*NSVT/VPCs* Non sustained ventricular tachycardia Ventricular premature complexes, *CHB / BBB* Complete Heart block/ Bundle branch block, *CMRI* Cardiac MRI, *N-terminal pro-B-type* natriuretic peptide, *LVEDP* Left ventricular end-diastolic pressure, *LV AO* Left ventricle to aorta gradient

### Cardiac MRI

**Fig. 1:**
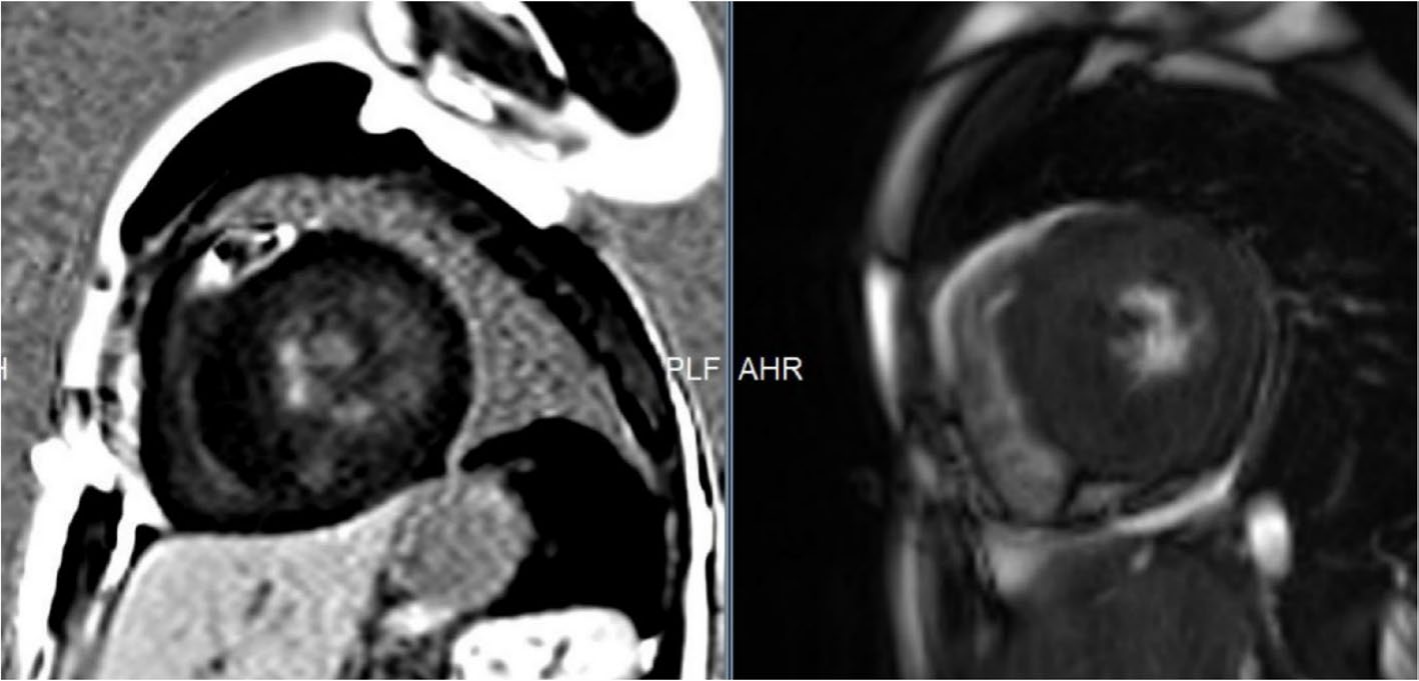
Short axis contrast-enhanced images of one of our HCM patients showing late gadolinium enhancement in lateral and inferior walls

Cardiac MRI was done in 28 patients, out of which 17 patients had Late gadolinium enhancement (60%). Table 6 shows the CMR characteristics of the cohort. Six had mid myocardial, three had subendocardial, and one had transmural enhancement. Four had patchy, and three had diffuse LGE. LGE was found in the septal wall of basal or midsegments in ten patients, the lateral wall in three patients, and the apex in five patients. We found LV wall thickness of more than 30 mm in 4 patients on CMR. Papillary muscle abnormalities were found in six patients and included accessory, bifid, and hypertrophied papillary muscles. Figure 1 shows a representative Cardiac MRI image of one of our HCM patients.

**Table 6:** Depicts the CMR characteristics of the twenty-eight children with HCM who underwent cardiac MRI

Age at CMR, yrs	12.9 ± 6.03
LVEF, %	68 ± 13.4
LVEDV, ml	93.27 ± 44.9
LVEDV indexed, ml/m^2^	69.45 ± 30.74
LVESV, ml	31.27 ± 29.37
LVESV indexed, ml/m^2^	23.85 ± 21.10
Stroke volume, ml	62.72 ± 29.23
Stroke volume indexed, ml/m^2^	45 ± 18.1
Maximum LV wall thickness, mm	25 ± 7.9
Maximum LV wall thickness indexed, mm/m^2^	18 ± 5.4
LV mass, g (*n* = 7)	241 ± 126
LV mass index, g/m^2^ (*n* = 7)	151.7 ± 71
LGE extent > 15%,n	4

*LV EDV* Left ventricle end diastolic volume, *LV ESV* Left ventricle end systolic volume, *EF* Ejection Fraction, *LGE* Late gadolinium Enhancement

Forty-eight patients were on a beta-blocker, mostly on metoprolol. Eight patients were on diuretics, one was on digoxin, and one was on ACE-Inhibitor. Two patients were on amiodarone, and one was on diltiazem. None of our patients were on Disopyramide.

### Outcomes

The outcome characteristics of the cohort are described in Table 7. Over a mean follow-up period of 5.6 years (0.1–30 years), twenty-one were lost to follow-up (33%). Among the patients whose outcome was known, eleven died (26%), and thirty-one (73%) were alive. The 5-year survival rate of HCM patients was 82%, and the 10-year survival rate was 78%. Out of the eleven patients who died, seven died of sudden cardiac death, three from heart failure, and one from ventricular arrhythmias. Sustained ventricular arrhythmias were seen in three patients and atrial tachyarrythmias in two. Both had atrial tachycardia with variable conduction, and none had atrial fibrillation. First-degree AV block was seen in 10 patients (15%) and bundle branch blocks (BBB) in five (8%). Eight patients required ICD or transplant (12.7%). Two patients underwent septal myectomy. One was a 13-year-old adolescent who underwent septal myectomy with mitral valve repair and RCA ostioplasty because of the associated slit-like RCA ostium. Another patient was one year old, a case of Noonan syndrome, who underwent septal myectomy with mitral valve replacement at 16 yrs age. Both achieved complete resolution of their outflow tract gradients and are alive and asymptomatic after 8 and 24 yrs of follow-up. Two patients underwent ICD implantation for primary prevention, one at 13 yrs of age who had a strong family h/o SCD and h/o syncope, and the other was 18 yr old with h/o recurrent syncope. Four patients underwent an electrophysiological study, two underwent to look for any inducible VT, one for pre-excitation and one for 2:1 AV Block, revealing distal AV conduction disease for which he underwent PPI.

**Table 7:** Outcome characteristics of HCM patients

Outcome (*n* = 63)	
Years of Follow up (mean)	5.6 (0.1–30)
Alive (% of pts on follow up)	31 (73)
Death (% of pts on follow-up)	11 (26)
Death from HF	3
SCD	7
Death from ventricular arrhythmias	1
Lost to follow up (%)	21 (33)
Sustained Ventricular arrhythmias, n(%)	3 (3)
Atrial arrhythmias, n(%)	2 (3)
AV block, n(%)	10 (15.8)
BBB, n(%)	5 (8)
Requirement of ICD/CRT/Transplant, n(%)	8 (12.7)
Septal myectomy, n	2 (Both asymptomatic, alive)
ICD/PPI, n	3 (2 underwent ICD for primary prevention, 1 underwent PPI for distal AV conduction disease)
EPS, n	4

*SCD* Sudden cardiac death, *AV block* Atrioventricular block, *BBB* Bundle Branch block, *EPS* Electrophysiological study, *ICD* Implantable cardioverter-defibrillator, *CRT* Cardiac Resynchronization Therapy, *PPI* Permanent pacemaker Implantation

The relationship between various clinical, echocardiographic and radiological risk factors and death or ventricular arrhythmias in the HCM cohort is depicted in Table 8. Only Consanguinity showed a trend towards higher events (*P*-value 0.08). Table 9 compares demographic characteristics of infantile-onset HCM with those with onset after the infantile period. Infantile onset was associated with higher mortality, though the *P*-value was insignificant (0.13).

**Table 8:** Factors affecting death/ventricular arrhythmias in HCM patients – univariate analysis

**Variable**	*P*-value
Age	0.13
Syncope	0.39
Family H/O Cardiomyopathy	0.49
Family H/O Sudden cardiac death	0.47
Consanguinity	0.08
QT Dispersion	0.27
IVS z score	0.68
LAE z score	0.40
SAM	0.75
NSVT on holter	0.70
LGE on MRI	0.26
LVOT obstruction	0.70

*LGE* Late gadolinium Enhancement, *LVOT* Left ventricle outflow tract, *NSVT* Non sustained Ventricular Tachycardia, *SAM* Systolic anterior motion, *LAE* Left atrial enlargement, *H/O* History of

**Table 9:** Compares the demographic characteristics of patients with infantile-onset HCM with those with onset after the infantile period

	Infantile onset (*n* = 23)	Non infantile onset (*n* = 40)
Gender, Males, n(%)	14 (60)	24 (60)
Family H/O HCM, n(%)	4 (17)	13 (34)
Family H/O SCD, n(%)	3 (13)	11 (27)
Consanguinity	3	2
Noonan Syndrome	4	5
Obstruction, n(%)	9 (39)	23 (57)
LGE	4 (*n* = 7)	13 (*n* = 21)
Mortality, n(%)	6 (26%)	5 (12%)
Cause of mortality		
SCD	3	4
HF	3	0
VA	0	1

*SCD* Sudden cardiac death, *HF* Heart Failure, *VA* Ventricular Arrhythmias

## Discussion

This is the most extensive study on pediatric cardiomyopathy from South Asia to our knowledge. We studied the clinical profile and outcomes of cardiomyopathy among children less than 18 years of age. Among the 233 cases identified, 63 cases had hypertrophic cardiomyopathy (27 percent). Although, classically, HCM was thought not to present in infancy, 12% of HCM patients presented in the neonatal period and 37% presented in the first year of life. Earlier studies did not specifically mention the percentage of cases diagnosed in the neonatal period. The median age of presentation of HCM patients in this study was seven years which is almost similar to the study by Lipschultz et al., who also reported a high incidence of HCM in infancy, and the median age of presentation of HCM patients was reported to be 5.9 years [12]. The high percentage of HCM cases presenting in infancy in our study may represent metabolic causes of HCM. Even though Nugent et al. excluded patients with multisystem metabolic causes of cardiomyopathy, they found more than 50% of HCM patients diagnosed in infancy and the median age of presentation of HCM patients to be unusually early, around 5.7 months [6]. This may represent the cases that have been diagnosed as a result of screening. The inclusion of metabolic causes of HCM may have underestimated survival as IEM is associated with worse survival among all etiological subtypes.

Among the 63 HCM patients, only sixteen patients had proven syndromic, metabolic, or genetic disease (25%). Nugent et al. found an underlying syndromal, genetic, or metabolic condition in 57.5% of subjects [1]. This may be because testing was not performed in most of the subjects because of non-availability or financial issues. In our study, LV outflow obstruction was present in 30 patients (47%), almost similar to the pediatric studies conducted so far. In the study by Nugent et al. [1], 40% of patients had LV outflow obstruction, and in the study by Yetman et al. [2], 59% had LV outflow obstruction. In adult HCM literature, LVOTO has been described in 75% either at rest or provocation [13]. Whether LVOT obstruction should be considered as a risk factor for sudden cardiac death remains debatable with some studies reporting that it is associated with a higher risk for SCD while as others have observed that it has a high negative predictive value for SCD [14, 15]. Only four subjects progressed to the burned-out phase in this series. Interestingly, three out of the four had non-obstructive HCM. This observation is similar to that found in other studies. Biagini et al. did not find LVOT obstruction in any of his cohort of burned out HCM [16]. The youngest age at which end stage phase was recognized was 8 yrs (Table 4). Though classically unfavorable adverse remodeling occurs after decades, it has been documented in patients as young as 5 yrs [9, 16].

One of our subjects with infantile-onset of obstructive HCM had infective endocarditis of the mitral valve at eight years of age. Fortunately, he responded to medical treatment. The patient had severe LA dilatation with an LA diameter z score of 5. Infective endocarditis is rare in adults with HCM and is limited to case reports in pediatric HCM [17]. Spirinto et al. found that endocarditis in HCM is virtually confined to the mitral valve and patients with outflow obstruction and is more common in those with both obstruction and atrial dilatation [18]. However, subsequent studies found similar rates of mitral and aortic valve involvement regardless of the presence of left ventricular outflow tract obstruction [19].

Noonan syndrome was present in 9 of the 63 patients with HCM (14%). Out of the nine patients, three presented in infancy (33%). Six of the nine patients were females. Two patients had associated pulmonary stenosis, and one underwent pulmonary balloon valvuloplasty for severe valvular pulmonary stenosis. The outcome was known in 5 out of the nine patients, and all are alive over a mean follow-up of 7 years. One underwent septal myectomy and is doing well after 24 years of follow-up. We have not compared the HCM patients with NS with those without, as our numbers are small. Wilkinson et al. compared data in 74 children with NS and HCM and 792 with idiopathic or familial isolated HCM [20]. Children with NS were diagnosed with HCM before six months old more often (51%) than children with HCM without NS (28%) and were more likely to present with congestive heart failure (24% vs. 9%). Patients with NS with HCM have a worse risk profile at presentation than other children with HCM, resulting in significant early mortality (22% at one year). One patient with MYBPC3 mutation-positive was a 15-year-old boy, a case of myoclonus dystonia with a family history of cardiomyopathy and sudden cardiac death who had diffuse subendocardial enhancement on C MRI. Myoclonus dystonia has never been previously described in MYBPC3 positive HCM patients. Preexcitation was present in two of our patients. First-degree AV block was present in 15% of HCM patients. Only three patients had sustained ventricular arrhythmias. Out of the 17 patients with LGE, four patients had LGE extent > 15%.

Over a mean follow-up period of 5.6 years (0.1–30 years), twenty-one were lost to follow-up (33%). Among the patients whose outcome was known, eleven died (26%), and thirty-one (73%) were alive. This gives 5-year survival rates of 82% and 10-year survival of 78%, which is comparable to other studies. Nugent et al. reported a 5-year survival of 83% [1], while Maruizi et al. reported a 5 yr survival of 95% [3]. Maruizi et al. excluded both syndromal and metabolic causes of HCM in their study, which might be the reason for improved survival in that cohort. Among the eleven patients who died, seven faced sudden cardiac death, three died from heart failure, and one died after recurrent ventricular arrhythmias. Sudden cardiac death has been reported to be the most common cause of death in adult HCM literature as well [21]. We acknowledge that fewer patients underwent SM than in other studies, despite 23 having a gradient > 50 mmHg on echocardiography, probably because most were not severely symptomatic after GDMT. Only one child was NYHA FC III despite GDMT and had a gradient of 100 mmHg on echocardiography and 60 mmHg on cardiac cath but refused invasive treatment. Although our numbers are small, we tried to analyze various postulated predictors of death or ventricular arrhythmias. None of the variables studied had a significant association with death or ventricular arrhythmias (Table 7). This may be due to the high percentage of patients lost to follow-up. Only Consanguinity showed a trend towards higher events of death or ventricular arrhythmias (*P*-value 0.08), though it did not reach statistical significance. This may reflect underlying inborn errors of metabolism which may have gone unrecognized. Norrish et al. [22] also noted that among the pediatric HCM patients, infantile onset and inborn errors of metabolism had the worst survival among all other etiological groups. Maurizi et al. [3] found that limiting symptoms at diagnosis and Troponin I and T gene mutations were associated with higher risk of lethal arrhythmic events. Interestingly, in his study, likelihood of lethal arrhythmic events was poorly related to classic adult risk factors like extreme LVH and syncope.

The study's primary limitation is the high attrition rate, which is not surprising in a retrospective study conducted over three decades. The difficulty of contacting patients who visited the hospital over the past three decades is obvious. Another limitation of the study is the lesser number of genetic and metabolic abnormalities identified as the testing was not available for most of the study period, and even now, few parents can afford the costs involved. Though it is a single-center study, it may be close to a population-based study as it is from a major referral center of the region. We also acknowledge that this cohort comprises comparatively lesser number of HCM patients than other studies, albeit the study has been conducted over three decades. This could be due to lack of systematic family screening in the early periods of the study.

## Conclusion

More than one-third of our HCM cohort presented in infancy. LV outflow tract obstruction is common (47%). Mid myocardial enhancement was the most common pattern of late gadolinium enhancement. SCD was the most common cause of death. The outcome in our HCM cohort is good and similar to other population cohorts. Only Consanguinity showed a trend towards higher events of death or ventricular arrhythmias, though it did not reach statistical significance.

## Data Availability

All data is available with the corresponding author and can be reproduced at any time.

## Abbreviations

HCM: Hypertrophic cardiomyopathy
LGE: Late gadolinium enhancement
LV: Left ventricle
MRI: Magnetic Resonance Imaging
ICD: Implantable Cardioverter Defibrillator
AV Block: Atrioventricular Block
BBB: Bundle Branch Block
SCD: Sudden Cardiac Death
NSVT: Non sustained Ventricular Tachycardia
LVH: Left Ventricular Hypertrophy
NS: Noonan Syndrome
LVOTO: Left ventricle Outflow Tract Obstruction
PPI: Permanent Pacemaker Implantation
GDMT: Guideline Directed Medical Therapy
NYHA: New York Heart Association
SM: Septal Myectomy
